# How Does Social Interaction Impact Grey Matter Volume in Healthy Older Women?

**DOI:** 10.1002/alz.084729

**Published:** 2025-01-09

**Authors:** Heather Kwan, Ashleigh F. Parker, Cassandra Szoeke, Jodie Gawryluk

**Affiliations:** ^1^ University of Victoria, Victoria, BC Canada; ^2^ University of Melbourne, Melbourne Australia; ^3^ University of Melbourne, Melbourne, VIC Australia; ^4^ Women’s Brain Project, Guntershausen Switzerland

## Abstract

**Background:**

The global population of adults over the age of 65 is expected to surpass 2 billion by 2050. Alongside this rise in the aging population, the incidence of age‐related cognitive decline and dementia will continue to grow. Importantly, women are at an elevated risk of cognitive decline compared to men. Therefore, it is imperative to identify modifiable risk and protective factors for aging women. The Lancet Commission highlighted low social contact as a modifiable risk factor for dementia and the Scaffolding Theory of Aging and Cognition‐Revised suggests potential neural structural benefits of such sources of enrichment. Structural neuroimaging allows for the examination of grey matter volume directly, in vivo. It was hypothesized that women with greater social interaction would demonstrate greater grey matter volume.

**Method:**

The current study used magnetic resonance imaging to examine the relationship between grey matter volume and social interaction in healthy older women. Participants were all biological females over the age 60 and obtained from the Women’s Healthy Ageing Project. The current study included 151 healthy older women (M_Age_ = 70.30 ± 2.88) who had 3T structural magnetic resonance imaging data and had completed the social composite score on the Short Form‐36 (SF‐36). The correlation between the social composite score and grey matter volume was examined using voxel‐based morphometry.

**Result:**

The results did not reveal significant correlations between grey matter volume and scores on the SF‐36, although there were sub‐threshold (*p*<0.2, corrected) positive correlations in the posterior areas of the brain. A post‐hoc region of interest analysis also revealed a significant correlation between SF‐36 and grey matter volume in the cerebellum (*p*<0.05, corrected).

**Conclusion:**

Age‐related cognitive decline continues to represent a significant and ongoing issue for our global population, particularly for women who are at an increased risk. It is crucial to continue to investigate the unique variables, such as social interaction, to improve the quality of life for aging women.

